# Supplementary material for: Multisensory stimuli facilitate low-level perceptual learning on a diﬃcult global motion task in virtual reality
Source: PLoS One. 2025 Mar 4;20(3):e0319007. doi: 10.1371/journal.pone.0319007 (PMC11878941; doi:10.1371/journal.pone.0319007)
Supplement: Attachment — Submitted filename: Response to reviewers.pdf [file pone.0319007.s001.pdf]

Dear Editor and Reviewers,

We thank you for your significant efforts over the course of the review process, and the positive impact they have had on the final manuscript. The final changes suggested were minor, and are reflected in the revised manuscript with blue text, as follows:

Reviewer #1:
Abstract: "specificity suggested" -> "specificity suggests"

Response: done

l. 43: "intact individuals" -> "healthy individuals"

Response: done

l. 259: "was statistically independent" -> "was independent"

Response: done

l. 308: "))" -> ")"

Response: done

p. 8, line 302 to p. 10, line 359: Use equal sign for exact p values (e.g., "p = .290") and the smaller sign only for p values that cannot be reported exactly because of too many decimals (e.g., "p < .001").

Response: done (but not colored blue)

However, there are a few changes we did not make:

- Reviewer #1 reported a typographical error in the abstract ("thanunisensory") that was not present in our version, or the version provided by the PLOS automated formatting system. *As such, we are unable to address this issue.*
- Reviewer #1: Caption of Fig. 4 (p. 9) and 2nd para of p. 10: The bars in Fig. 4 are actually red and blue and not orange and purple. Please make it consistent either by correcting the text or the colors in Figure 4.
  This was not a mistake - we surmise it may have been a perceptual differences caused by differences in display properties, a color-space conversion problem, the heavy image compression used in generation of the PLOS preprint, or other factors. After some deliberation with colleagues, we have changed the text to indicate blue bars but have left references to the orange bars unchanged. Because there are no other bars that could be confused as either red or orange, we do not think this potential ambiguity will affect interpretability. We will continue to monitor this situation through the PDF conversion process during the final stages of submission.
